# Structural Models for Roseolovirus U20 And U21: Non-Classical MHC-I Like Proteins From HHV-6A, HHV-6B, and HHV-7

**DOI:** 10.3389/fimmu.2022.864898

**Published:** 2022-04-04

**Authors:** Grant C. Weaver, Richa Arya, Christine L. Schneider, Amy W. Hudson, Lawrence J. Stern

**Affiliations:** ^1^Immunology and Microbiology Graduate Program, Morningside Graduate School of Biomedical Sciences, UMass Chan Medical School, Worcester, MA, United States; ^2^Department of Pathology, UMass Chan Medical School, Worcester, MA, United States; ^3^Department of Life Sciences, Carroll University, Waukesha, WI, United States; ^4^Department of Microbiology and Molecular Genetics, Medical College of Wisconsin, Milwaukee, WI, United States; ^5^Department of Biochemistry and Molecular Biotechnology, UMass Chan Medical School, Worcester, MA, United States

**Keywords:** human herpesvirus, major histocompatibility protein, immunoevasion, machine learning, structure prediction, MHC1b, natural killer cell ligand, immune recognition

## Abstract

Human roseolovirus U20 and U21 are type I membrane glycoproteins that have been implicated in immune evasion by interfering with recognition of classical and non-classical MHC proteins. U20 and U21 are predicted to be type I glycoproteins with extracytosolic immunoglobulin-like domains, but detailed structural information is lacking. AlphaFold and RoseTTAfold are next generation machine-learning-based prediction engines that recently have revolutionized the field of computational three-dimensional protein structure prediction. Here, we review the structural biology of viral immunoevasins and the current status of computational structure prediction algorithms. We use these computational tools to generate structural models for U20 and U21 proteins, which are predicted to adopt MHC-Ia-like folds with closed MHC platforms and immunoglobulin-like domains. We evaluate these structural models and place them within current understanding of the structural basis for viral immune evasion of T cell and natural killer cell recognition.

## Introduction

**Figure 1:**
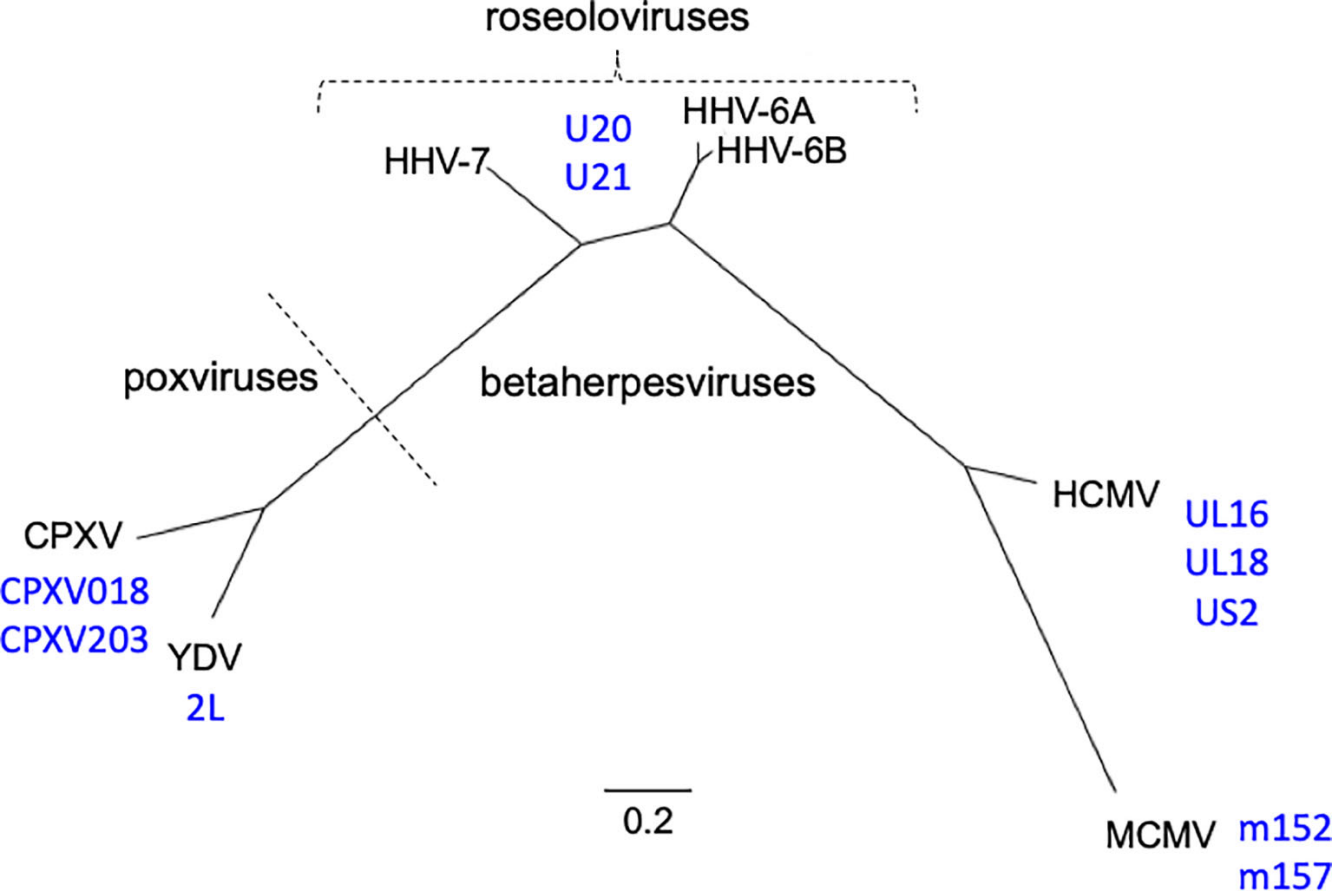
Phylogenetic relationships of viruses and viral immunoevasins discussed in this work. The complete genomic DNA sequences from HHV-6A (strain U1102), HHV-6B (strain Z29), HHV-7 (strain JI), HCMV (strain HAN-SCT17), and MCMV (strain N1) and poxviruses Cowpox (strain Brighton Red) and Yaba-like Disease Virus (strain Amano) were retrieved from the VIPR database (**Table 1**) and aligned using the MEGA11 software package. MEGA was then used to generate a maximum likelihood phylogeny reconstruction (1). Scale bar represents the probability of a nucleotide substitution at a given site, calculated for select betaherpesviruses. Viral MHC-like proteins that contribute to evasion of host T cell and NK cell responses and are discussed in this work are indicated next to the virus labels.

The roseolovirus genus of the β-herpesvirus subfamily includes Human Herpesviruses 6A, 6B, and 7 (HHV-6A, HHV-6B, and HHV-7) (**Figure 1**). These viruses have extremely high prevalence and infect over 90% of the world’s population before the age of 6 (2–4). While all three viruses primarily target activated T cells, there are some key differences in the range of additional cell types in which each virus can be found, with HHV-6A having a broader cell tropism compared to HHV-6B and HHV-7. Primary infection with these viruses in infancy causes exanthem subitum, commonly known as roseola or sixth disease, with fever, rash, and occasionally febrile seizures (5). While primary infection rarely causes severe symptoms in immunocompetent individuals (6), like other herpesviruses the roseoloviruses establish lifelong latency with periodic reactivation. In immunocompromised individuals, roseoloviruses can cause severe complications, such as encephalitis and pneumonitis. Roseoloviruses are particularly problematic in solid organ transplant and hematopoietic stem cell transplant patients, where infection/reactivation can result in organ rejection and graft vs. host disease (4, 7, 8).

**Table 1:** Viral genomes referenced in this study.

Virus	Strain	Genbank ID
Human Herpesvirus 6A (HHV-6A)	U1102	NC_001664
Human Herpesvirus 6B (HHV-6B)	Z29	MW536483
Human Herpesvirus 7 (HHV-7)	JI	U43400
Human Cytomegalovirus (HCMV)	HAN-SCT17	MT649470
Mouse Cytomegalovirus (MCMV)	N1	HE610454
Yaba-like disease Virus (YDV)	Amano	NC_005179
Cowpox Virus	Brighton Red	NC_003663

Roseolovirus genomes are composed of linear, double stranded DNA that contains a unique (U) region flanked by a set of identical direct repeat regions (DR), which facilitate integration into the telomeric regions of chromosomes. For HHV-6A and -6B this can occur in the germ line, resulting in inherited chromosomally integrated HHV-6 in approximately 3% of the population (9, 10). The HHV-6A/B genomes are 160-170 kb while the HHV-7 genome is slightly smaller at 145-153 kb, although both contain between 100 and 120 ORFs. Overall, the amino acid identity between HHV-6A and HHV-6B is approximately 90% while the difference between HHV-7 and HV-6A/6B is closer to 50%. In order to facilitate lifelong latency, many of these genes encode products that allow the virus to modulate the host immune response. Much previous work with human and mouse cytomegaloviruses (HCMV and MCMV) and certain poxviruses including cowpox virus (CPVX) and Yaba-like disease virus (YLDV) has revealed some of the broad strategies that dsDNA viruses use for immune evasion. HCMV encodes as many to 40 gene products that can antagonize the immune system [reviewed in (11)] but many of the basic strategies are shared with other, similar viruses. Despite their dissimilarity at a genetic level, betaherpesviruses and poxviruses have evolved gene products that contain MHC-like and/or immunoglobulin-like domains that utilize similar strategies to attack the host immune response (**Figure 1**).

A common immune evasion strategy utilized by herpesviruses and some poxviruses is downregulation of classical MHC-Ia proteins to prevent presentation of viral antigens to CD8+ T cells. Since this activity can trigger NK cell “missing self” recognition, these same viruses often antagonize NK cell responses by interacting with activating and inhibitory NK receptors or their ligands, which in many cases are non-classical MHC-Ib stress-induced proteins. Viral homologues of nonclassical class I MHC molecules (vMHCs) play key roles in this process. Although these vMHCs have a diverse range of functions they all share the characteristic class I MHC fold [reviewed in (12–14)]. Roseoloviruses HHV-6A, HHV-6B, and HHV-7 all have been shown to downregulate host-derived cellular MHC-Ia (cMHC-Ia) (15, 16) and cellular MHC-Ib (cMHC-Ib) molecules (17–20) with the roseolovirus U21 glycoprotein responsible for this activity by intercepting cMHC-Ia in the ER and directing it to lysosomes for degradation (21, 22), and the U20 protein recently implicated in MHC-Ib downregulation (18). However, many questions remain surrounding the structures and molecular mechanisms of these two proteins. In this article, we evaluate structural models for roseolovirus U20 and U21 proteins produced by next-generation computational machine learning approaches AlphaFold (23) and RoseTTAFold (24). These models provide insight into possible roles of U20 and U21 in roseolovirus immune evasion as non-classical MHC-Ib proteins.

## Viral Evasion of T Cell and NK Cell Recognition

**Figure 2:**
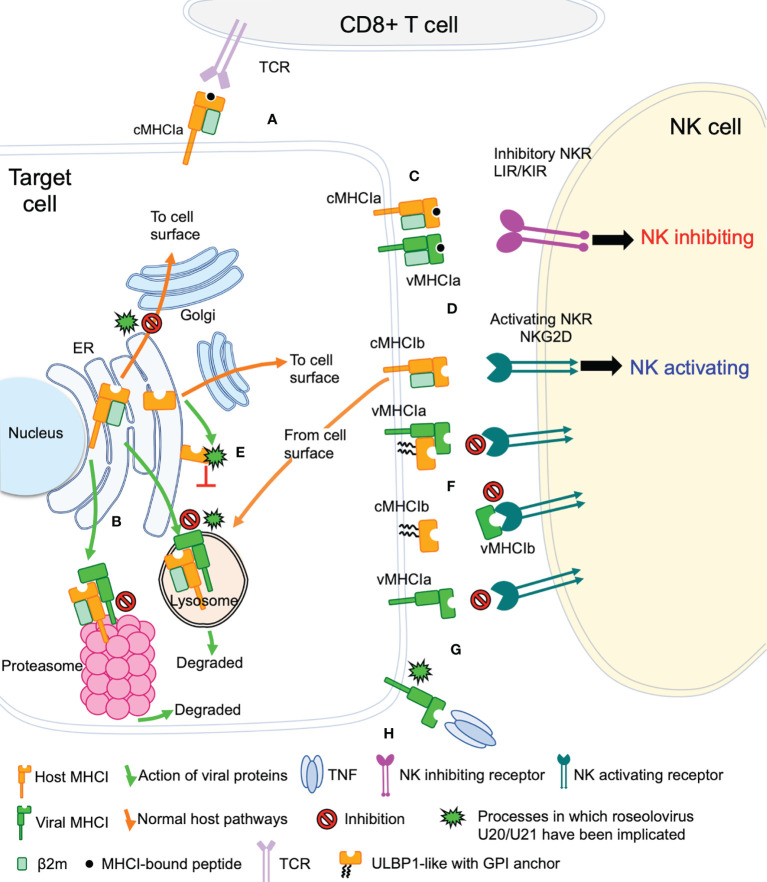
Viral evasion of T cell and NK cell recognition. Viral MHC-based immune evasion pathways employed by betaherpesviruses and poxviruses are shown. MHC-Ia refers to classical polymorphic peptide-binding class I MHC molecules, MHC-Ib refers to non-classical non-polymorphic class I MHC homologs that do not present peptides. cMHC-Ia and cMHC-Ib refer to cellular (host) protein, vMHC-Ia and vMHC-Ib refer to viral homologs. Other mechanisms employed by betaherpesviruses and poxvirus to evade T cell and NK cell recognition involve interference with antigen processing, transcriptional regulation, and innate immune evasion, but those pathways are not known to involve vMHC-I molecules and are not included here. **(A)** cMHC-Ia present peptides to CD8+ T cells, triggering induction of cytolytic pathways upon T cell receptor (TCR binding). **(B)** vMHC-Ib block this pathway by redirecting cMHC-Ia to proteasomes or lysosomes for destruction. **(C)** Inhibitory natural killer cell receptors (NKR) can recognize the loss of surface cMHC-Ia as part of the “missing-self” recognition system. vMHC-Ia combat this by engaging inhibitory NKR. **(D)** NK cells sense cellular stress using activating NKR to recognize stress-induced cMHC-Ib proteins. **(E)** vMHC-Ib block this pathway by redirecting cMHC-Ib to proteasomes or lysosomes for destruction. **(F)** vMHC-Ib can block activating NKRs by either masking them on the surface or by secreting vMHC-Ib variants that can engage activating NKR and competitively block recognition of cMHC-1b stress ligands. Wavy lines indicate glycosylphosphatidylinositol (GPI) membrane anchors used by some ULBP proteins instead of transmembrane helices. **(G)** Paradoxically, vMHC-Ib can engage activating NKR for reasons that remain poorly understood. **(H)** Another vMHC-Ib binds the inflammatory cytokine TNFα, sequestering it from productive engagement with TNF-receptors.

The game of evolutionary cat and mouse between virus and host has established a complicated back and forth evolution of viral and host gene products. Cytotoxic CD8+ T cells recognize viral peptides presented by host cell classical MHC-I molecules (cMHC-Ia) and respond by killing the infected cell (**Figure 2A**). To combat this, viruses evolved the ability to redirect MHC-I from the ER or cell surface to proteasomes or lysosomes for degradation, preventing T cell recognition and killing (**Figure 2B**). However, NK cells have evolved mechanisms that allow detection of the absence or reduction of MHC-I on the cell surface. In this pathway, inhibitory NK receptors, such as those in the LIR/KIR family, engage cMHC-Ia to generate suppressive signals that block NK activation (**Figure 2C**). To circumvent this, some viruses express homologs of classical MHC-I (vMHC-Ia), that mimic cMHC-Ia by engaging inhibitory NK receptors (**Figure 2C**). Another host defense mechanism involves the surface expression of stress-induced ligands for activating NK receptors, such as those in the NKG2D family (**Figure 2D**). In general, ligands for these activating NK receptors are non-classical MHC-I proteins (cMHC-Ib), which do not present peptide or other antigens. Structurally cMHC-Ib proteins are similar to classical cMHC-Ia proteins, although without an antigen binding groove, and in many cases structural elements such as the small β2-microglobulin subunit or lower immunoglobulin-like domain are missing. To evade this mechanism, viruses have evolved mechanisms to bind these activating NK ligands in the ER or at the cell surface and sequester them and/or divert them to the lysosome or proteasome for degradation (**Figure 2E**). In many cases these mechanisms involve vMHC-Ib molecules. Other vMHC molecules inhibit activating NK receptors by alternative mechanisms. One mechanism involves secretion of a soluble vMHC-Ib protein that acts as a decoy, competitively inhibiting activating NK receptor signaling (**Figure 2F**). Another involves a cell-surface vMHC-Ib, that paradoxically binds an activating NK receptor apparently without triggering signaling, the molecular basis of which remains unelucidated (**Figure 2G**). Finally, many viruses have evolved decoy receptors or ligands that interfere with cytokine signaling; in one case this involves a vMHC-Ib molecule capable of binding and sequestering TNFα, attenuating host immune responses to viral infection (**Figure 2H**).

## Structural Aspects of Classical and Non-Classical MHC Immunoevasion

**Figure 3:**
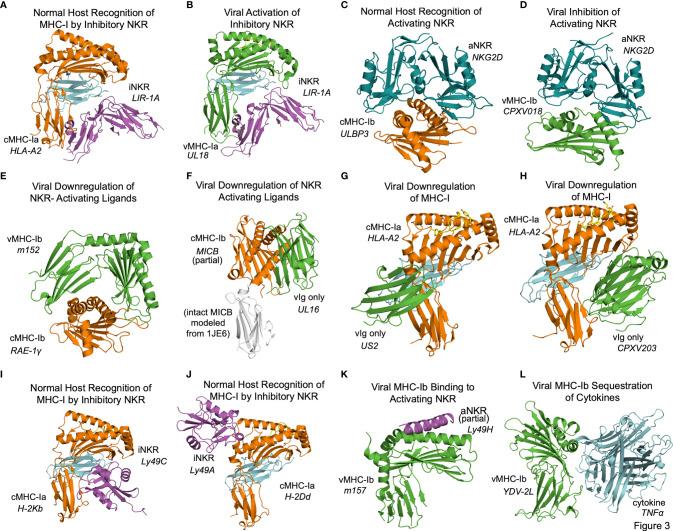
Structural aspects of classical and non-classical MHC immunoevasion. Crystal structures of cMHC-I (orange) and vMHC-I (green) in complex with cellular binding partners (**Table 2**). **(A)** cMHC-Ia protein HLA-A2 bound to inhibitory NKR LIR-1A (PDB: 1P7Q). **(B)** viral cMHC-Ia homolog UL18 from HCMV bound to inhibitory NKR LIR-1A (PDB: 3D2U). **(C)** cMHC-Ib ULBP3 bound to the activating NKR NKG2D (cyan) (PDB: 1KCG). **(D)** ULBP homolog CPXV018 from cowpox virus interacting with activating NKR NKG2D (cyan) (PDB: 4PDC). **(E)** m152, a vMHC-Ib protein bound to cMHC-Ib RAE-1γ, promoting retention and eventual degradation (PDB: 4G59). **(F)** vMHC-Ib UL16 from HCMV bound to cMHC-Ib MICB (PDB: 2WY3 and 1JE6). **(G)** Viral Ig-domain protein US2 from HCMV binding cMHC-Ia HLA-A2, targeting it for degradation (PDB: 1IM3). **(H)** Viral Ig-domain protein CPXV203 from cowpox virus binding cMHC-Ia HLA-A2, targeting it for degradation (PDB: 4HKJ). **(I)** cMHC-Ia protein H-2Kb bound to inhibitory NKR Ly49C (38CK). **(J)** cMHC-Ia protein H-2Dd bound to inhibitory NKR Ly49A (PDB: 1QO3). **(K)** vMHC-Ib m157 from HCMV interacting with activating NKR Ly49H (PDB: 4JO8). **(L)** vMHC-Ib 2L protein from YDL virus interacting with cytokine TNFα (PDB:3IT8).

In some cases the structural basis for vMHC-I-based immunoevasion is well understood. Certain vMHC-I directly engage inhibitory NK receptors similarly to the normal host ligands. The human LIR-1A inhibitory NK receptor binds to the canonical host cMHC-Ia HLA-A2 underneath the MHC platform domain (**Figure 3A** PDB: 1P7Q) (25). UL18 is a classical class I MHC homolog expressed by HCMV that mimics cMHC-Ia by binding LIR-1 in the same manner (**Figure 3B**, PDB: 3D2U) (26). Similarly to cMHC-Ia, UL18 binds both peptides and soluble β-2-microglobulin (β2m), making it a vMHC-Ia, but it is the only viral MHC homolog known to do so (26). Despite its ability to bind inhibitory NK receptors, the precise role of UL18 remains mysterious, as it has been shown to inhibit LIR-1 positive NK cell activation while promoting activation of LIR-1 negative NK cells (27, 28).

Other frequent targets of viral immunoevasins are activating NK receptors such as NKG2D. NKG2D binds to a variety of host cMHC-Ib ligands upregulated in cases of cellular stress or damage, including human ULBP and MIC family members, and the mouse RAE-1 family of ULBP homologs. NKG2D engages these receptors by forming a dimer that associates with the top of the MHC fold as illustrated by the ULBP3-NKG2D (**Figure 3C**, PDB: 1KCG) (29). Cowpox virus has evolved an interesting evasion mechanism where the vMHC-Ib protein CPXV018 (OMCP) binds the activating NK receptor NKG2D in a configuration very similar to native cMHC-Ib ligands (**Figure 3D**, PDB: 4PDC). Because the viral version is soluble instead of membrane-bound, it acts as a competitive inhibitor, with a 14-fold higher affinity for mouse NKG2D as compared to one of its typical ligands, RAE-1ϵ. This marked increase in affinity was attributed to a single loop that reaches up into the NKG2D binding pocket (30).

Another, less direct means of inhibiting NKG2D and NK activating receptors is to downregulate the activating cMHC-Ib ligands in the host cell. The MCMV m152 glycoprotein is a vMHCI-b with several important immunoregulatory functions that has been shown to bind and downregulate multiple members of the NK activating RAE-1 family. It contains an MHC platform domain and immunoglobulin-like domain, but not β2-microglobulin or peptide, and binds its targets in a claw-like manner, reaching over the top of the NK ligand’s MHC domain (**Figure 3E**, PDB: 4G59) (31). When this occurs in the ER, the target cMHCI-b is retained and subsequently degraded. Recently, m152 has been shown also to reach the cell surface and mask its RAE-family binding partners from being recognized by NKG2D, as well as affect IRF signaling through STING (32, 33).

Other viral immunoevasins that lack MHC platform domains down-regulate cMHC-Ia and cMHC-Ib through interactions with their immunoglobulin-like domains. The HCMV protein UL16 resides primarily in the ER and cis-Golgi, and downregulates NK ligands MICB, ULBP1, and ULBP2 by binding and sequestering them within the secretory system (34, 35). UL16 is a single immunoglobulin-like domain protein that engages MICB by binding perpendicularly to the α1 and α2 helices of the MHC platform domain and but parallel to the sheets of the MHC domain underneath (**Figure 3F**, PDB: 2WY3 and 1JE6) (36, 37). As with m152, the result of this interaction is that NKG2D activating ligands are sequestered in the ER and degraded. US2 is another single immunoglobulin-like domain protein that binds to host classical class I MHC proteins as they are synthesized in the ER, inducing them to be degraded (38). US2 binds underneath cMHC-Ia platform to the C-terminus of the α2 helix and the α3 domain (**Figure 3G**, PDB: 1IM3). Interestingly this interaction is similar to that of the inhibitory NK receptor Ly49A binding to cMHC-Ia (**Figure 3A**). Cowpox virus CPXV203, with similar structure and function to US2, binds on the opposite side of the host MHC-I in a manner similar to that of LIR-1 and other NK receptors, making contact with the MHC platform and β2m (**Figure 3H**, PDB: 4HKJ) (39).

In some cases, the interplay of host response and viral evasion protein evolution can be complex. This is exemplified by the Ly49 family of murine NK receptors. The Ly49 receptors can be either activating or inhibitory, and use C-type lectin domains to interact with MHC-Ia molecules [reviewed in (40, 41)]. Structures of inhibitory Ly49C and Ly49A family members bound to their normal MHC ligands reveal significant diversity in binding modes (**Figure 3I**, PDB 38CK and **Figure 3J** 1QO3) (42, 43). No vMHC proteins have been identified that bind inhibitory NK receptors using these particular modes of interaction, but these structures could provide models for viral immunoevasins yet to be characterized. The m157 glycoprotein of MCMV engages inhibitory NK receptors such as Ly49I to prevent missing-self recognition (44). Although detailed structural information is not available, mutagenesis suggests a different binding mode than for other Ly49 family inhibitory NKR engaging their host cMHC-Ia ligands (43, 44). There is a high degree of m157 sequence variability across MCMV strains, and m157 exhibits varying effects depending on the combination of mouse and virus strains. In fact, m157 from the Smith strain of MCMV binds the inhibitory NK receptor Ly49I in 129/J mice, but in C57BL/6 mice, m157 binds the activating NK receptor Ly49H (45). Although only a small part of Ly49H was visualized in the m157 complex structure, binding would appear to involve the top of the MHC platform (**Figure 3K**, PDB: 4JO8) (46). This interaction contributes to NK activation and viral clearance, and it has been hypothesized that Ly49H has evolved to recognize m157 to thwart NK evasion by MCMV (46).

Finally, the Yaba-like Disease Virus 2L protein has an MHC-like fold that binds TNFα in a manner different from other viral TNFR mimics to achieve picomolar binding affinities that allow it to compete quite effectively with the host receptor (47) (**Figure 3L**, PDB: 3IT8).

## U20 and U21 From HHV-6A, HHV-6B, HHV-7

**Figure 4:**
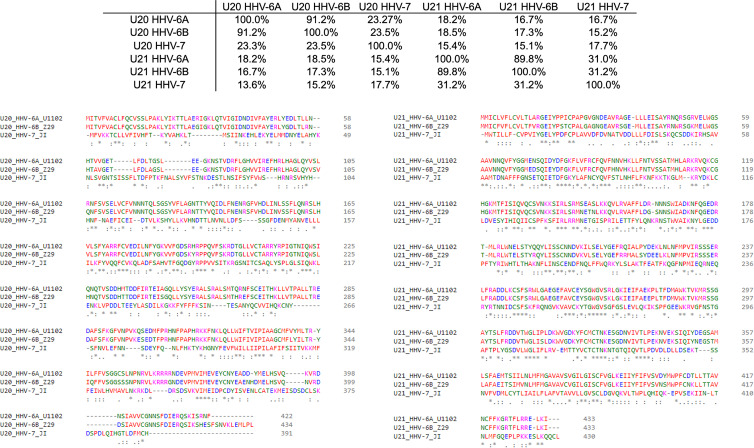
Sequence homology of U20 and U22 from HHV-6A, HHV-6B, HHV-7. The amino acid sequences for U20 and U21 from HHV-6A, -6B, and -7 were retrieved from the Uniprot database (**Table 3**). They were then aligned using Clustal Omega (49). Top: Sequence identity matrix for U20 and U21 from HHV-6A, HHV-6B, and HHV-6. Bottom Left: Alignment of U20 sequences from HHV-6A (strain U1102), HHV-6B (Z29) and HHV-7 (JI). Amino acids are colored by type. Identities are indicated by an asterisk and similarities are indicated with a colon. Bottom Right: Alignment of U21 sequences from these same strains, colored and annotated as in B.

The complex interplay between host and viral immunomodulators has been best characterized in MCMV and HCMV and is just now beginning to come into focus for the roseoloviruses. The first pieces of the puzzle fell into place when it was shown that U21 can downregulate non-classical cMHC-Ib stress-response proteins in addition to classical cMHC-Ia proteins. In HHV-7, U21 was shown to bind and downregulate the nonclassical MHCs HLA-E and HLA-G, as well as the NK activating ligands MICA and MICB (19, 20). Also, U21 expressed by HHV-6A is able to downregulate ULBP3 (18). In HHV-6B, the precise role of U21 has yet to be defined, but it is clear that the NK ligands ULBP1, ULBP3, and MICB are downregulated during infection (17). Very recently, HHV-6A U20 was shown to downregulate ULBP1 from the cell surface, decreasing NK activation in degranulation assays (18). In addition, U20 from HHV-6B has been shown to affect TNFR signaling, inhibiting PARP cleavage, caspase 3 and 8 activation, and IκBα and NF-κB transcriptional activity (48). Although roles for HHV-6 U20 and U21 in down-regulating cMHC-Ia and cMHC-Ib have been identified, several questions remain. For example, based on the high degree of sequence conservation (91.2%) between the amino acid sequences of U21 among HHV-6A and -6B (**Figure 4**), one would expect that U21 likely plays a similar role in both viruses. However, HHV-6A U21 seems to be more efficient at downregulating MHC-I as compared to HHV-6B U21 (22). Additionally, U21 from the various roseoloviruses exhibit the ability to bind variable targets from one virus to another despite a high degree of conservation (15, 19, 20). Finally, while it has been proposed that U21 might adopt an MHC-like fold (50), there is not yet structural evidence to support this and even less is known about the structure of U20. U21 redirects and sequesters its targets in the ER where they are eventually redirected to the lysosome (21). However, U20 accomplishes ULBP1 downregulation by an alternative, lysosome-independent mechanism that remains to be elucidated (18). To better understand how these roseolovirus immunoevasins fit into the picture of T cell and NK cell evasion presented above, we utilized advanced structure prediction tools to generate structural models and draw parallels to the better-characterized viral immunoevasins just discussed.

**Table 2:** Classical and nonclassical MHC structures referenced in this study.

Protein Name(s)	Uniprot Accession ID	PDB ID
US2 + HLA-A2	P09713, P04439	1IM3
MICB	Q29980	1JE6
ULBP3 + NKG2D	Q9BZM4, P26718	1KCG
HLA-A2 + LIR-1	P04439, Q8NHL6	1P7Q
H-2Dd + Ly49A	P01900, P20937	1QO3
UL16 + MICB	P16757, Q29980	2WY3
H2-Kb + Ly49C	P01901, Q64329	3C8K
UL18 + LIR-1	P08560, Q8NHL6	3D2U
2L + TNF	Q9DHW0, P01375	3IT8
m152 + RAE-I	Q83156, O08604	4G59
CPXV203 + HLA-A2	Q8QMP2, P01901	4HKJ
m157 + Ly49H	Q6XK91, Q60682	4JO8
CPXV018 + NKG2D	Q8QN43, P26718	4PDC

**Table 3:** Roseolovirus protein sequences referenced in this study.

Protein Name	Uniprot Accession ID
U20 (HHV-6A)	Q69555
U20 (HHV-6B)	Q9QJ46
U20 (HHV-7)	Q69502
U21 (HHV-6A)	Q69556
U21 (HHV-6B)	Q9QJ45
U21 (HHV-7)	P60505
U22 (HHV-6B)	A0A894YVT6
U23 (HHV-6B)	A0A219Y0C2
U24 (HHV-6B)	Q69559
U25 (HHV-6B)	Q9WT39
U26 (HHV-6B)	A0A650BQA3

## Evolution of HHV-6B U20 Structure Prediction

**Figure 5:**
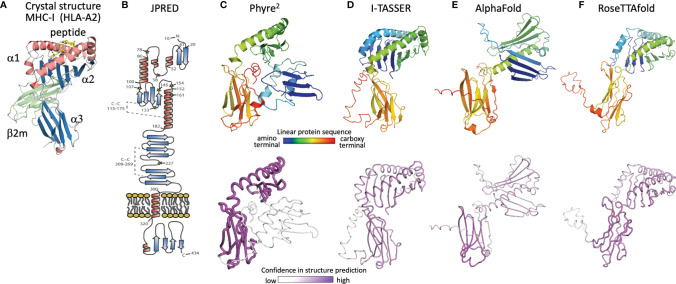
Evolution of U20 structure prediction. **(A)** Structure of HLA-A2, a classical MHC-Ia protein that adopts the canonical MHC fold. MHC α1 and α2 domains in the MHC platform and α3 immunoglobulin-like domain colored by secondary structure for comparison with panel B, with the MHC-associated non-polymorphic small subunit β2-microglobulin shown in green and bound peptide shown in yellow. **(B)** Secondary structure prediction from JPRED (48, 53), figure reproduced with permission from reference (48). **(C–F)**, Three-dimensional structure predictions from **(C)**, Phyre^2^, **(D)**, I-TASSER, **(E)**, AlphaFold, and **(F)**, RoseTTAfold. Top panels show ribbon diagrams colored by linear protein sequence; bottom panels show tube diagrams colored by modeling confidence.

With no experimental characterization of U20 or U21 structures, computational tools can be used to gain insight into their structures and potential binding partners. We first review the evolution of computational structure prediction for these proteins, using U20 from HHV-6 as an example. The presence of immunoglobulin-like domains can be robustly predicted from sequence data alone, using the pattern of hydrophobic, hydrophilic, and turn-inducing amino acid residues along with a presence of the characteristic disulfide-bond linking the two component beta sheets (51). An immunoglobulin-like fold was predicted for HHV-6B U20 along with the initial genomic sequence (52). This prediction was further supported by analysis of predicted secondary structure elements, which also can be predicted from raw sequence data using JPRED or similar algorithms (53). The C-terminal half of the extracellular region shows the typical pattern of disulfide-linked 3-stranded and 4-stranded beta sheets (**Figure 5B**), as expected for membrane-proximal immunoglobulin-like domain and reported by Kofod-Olsen et al. (48). Interestingly, the N-terminal half of the extracellular region shows a pattern of predicted sequential beta strands followed by alpha-helical regions. This pattern is characteristic of the MHC-I fold, which is composed of an eight-stranded beta sheet topped by two alpha helices, sitting above an immunoglobulin C-type domain. This is illustrated for the classical MHC-Ia protein HLA-A2 (**Figure 5A**), represented as a ribbon diagram and colored for correspondence with the JPRED secondary structure diagram. The pattern of two sequential copies of a strand-strand-strand-strand-helix motif characteristic of the MHC platform fold was used in the original discovery of non-classical MHC-Ib proteins from MCMV as ligands for NK receptors (54). The computational structure prediction engine used in that work was 3D-PSSM, which matches predicted secondary structure patterns in the target sequence with similar patterns present in proteins with known three-dimensional structures. The use of protein-specific scoring matrices and hidden Markov models in 3D-PSSM was an early application of machine learning to protein structure prediction (55). In 2009 we used Phyre (56), a successor to 3D-PSSM, to extend that approach to HHV-6B U20, but an MHC platform domain was not identified, although the immunoglobulin domain was robustly detected. An improved version of Phyre, Phyre^2^, considers sequence-based predictions of disordered regions as well as secondary structures, and aligns homologous sequences prior to secondary structure prediction for more robust pattern detection (57). The Phyre^2^ prediction for HHV-6B U20 shows a four stranded beta sheet and alpha helix atop a canonical immunoglobulin-like domain (**Figure 5C**). A second beta sheet with associated alpha helix, N-terminal to the one just mentioned, is present but not assembled into the canonical fold (**Figure 5C**) and is predicted with much lower confidence. The PHYRE^2^ modeling quality score is shown as a tube model, with the magenta intensity and tube radius increasing with proportionally to confidence in structure prediction (**Figure 5C** bottom). We also predicted a three-dimensional structure for HHV-6B U20 using I-TASSER, a more recent structure prediction engine (**Figure 5D**). I-TASSER uses a threading alignment refinement procedure, which aligns target sequences onto known three-dimensional structure templates (“threading”), and then evaluates energetics and steric clashes for local regions of three-dimensional space. Low-energy local regions are clustered and assembled into domains, and the process is iterated (58, 59). Models are scored using the root mean square deviation (RMSD) of structures used in the clustering and a TM-score representing the differences between pairwise distances for all atoms in local region between models in the cluster, which provides an improved per-residue confidence score. The I-TASSER model for HHV-6B U20 shows both halves of an assembled canonical MHC-I like domain, with similar confidence throughout (**Figure 5D**). U21 has also been previously predicted to adopt an MHC-like fold based on a similar analysis using Phyre^2^ and I-TASSER, with similar results for the HHV-6A, HHV-6B, and HHV-7 orthologs of U20 and U21 (50).

The protein structure prediction community was rocked in 2018 by the performance of AlphaFold (60), a machine learning algorithm from Google’s DeepMind project, which also produced the chess- and GO-playing AlphaZero and AlphaGo algorithms (61). Since 1994, protein modelers have participated in the biennial Critical Assessment of Protein Structure Prediction (CASP), a competition to predict recently determined but unreleased protein structures (62). Sustained progress has been made in each cycle (Phyre, Phyre^2^ and I-TASSER were prominent among the top scoring computational algorithms from CASP 8 in 2012 to CASP-12 in 2016). CASP-13 saw an unprecedented increase in prediction accuracy, with DeepMind’s AlphaFold demonstrating more than 20% increased accuracy of backbone prediction for the most challenging structure prediction category, proteins with marginal similarity to any known structure (63). In 2020 at CASP-14 an improved AlphaFold algorithm continued this trend, dramatically outperforming other strictly computational as well as human-assisted approaches, with precision in some cases approaching expected experimental error (23). AlphaFold employs parallel tracks of one-dimensional multiple sequence alignments, two-dimensional patterns of pairwise co-evolution of residues in homologous sequences, and three-dimensional structural representations that minimize distance between co-evolving pairs of residues, with machine-learning optimizations in each track and iterative propagation of information between tracks. A novel aspect is that the three-dimensional structure is modeled initially as an “atomic gas” of interacting residues without conventional protein structure constraints such as bond lengths and angles, torsions, electrostatics, and conformational constraints, which are applied only subsequently during conventional gradient-descent refinement and Amber force field after the end-to-end ab initio structure determination is completed. AlphaFold also introduced an improved confidence measure, the predicted local Cα distance difference test (pLDDT). Similar approaches were incorporated into RoseTTAfold, an effort by prominent academic protein structure groups to make a similar but somewhat simplified algorithm available to the larger community, on an open-source platform suitable for use on conventional computer hardware (24).

The AlphaFold prediction of HHV-6B U20 structure includes the components of the conventional MHC-I fold, but with the domains reoriented (**Figure 5E**). The MHC platform α1 and α2 domains are displaced from each other and from the immunoglobulin-like α3 lower domain, and the α1 helix is largely missing. Modeling confidence is higher for the immunoglobulin domain than for the platform domains, with the α2 confidence score low but still somewhat higher than for α1. Notably the platform domains are oriented “upside-down” relative to a conventional MHC-Ia structure, somewhat similarly to the arrangement in certain MHC-Ib proteins like MICA and MICB, but with an even more extreme displacement, and the α1 domain strand threading pattern is different (DABC versus ABCD). In contrast, RoseTTAfold predicts a more canonical MHC-like fold, with conventionally threaded and oriented α1 and α2 domains, which however still are considerably displaced from the α3 immunoglobulin-like domain (**Figure 5F**). Modeling confidence is similar for the α2 and α3 domains and only slightly lower for α1, although RoseTTAfold reports an RMSD-based score which is believed to be less accurate than and not directly comparable to pLDDT (64).

## U20-U26 Gene Cluster

**Figure 6:**
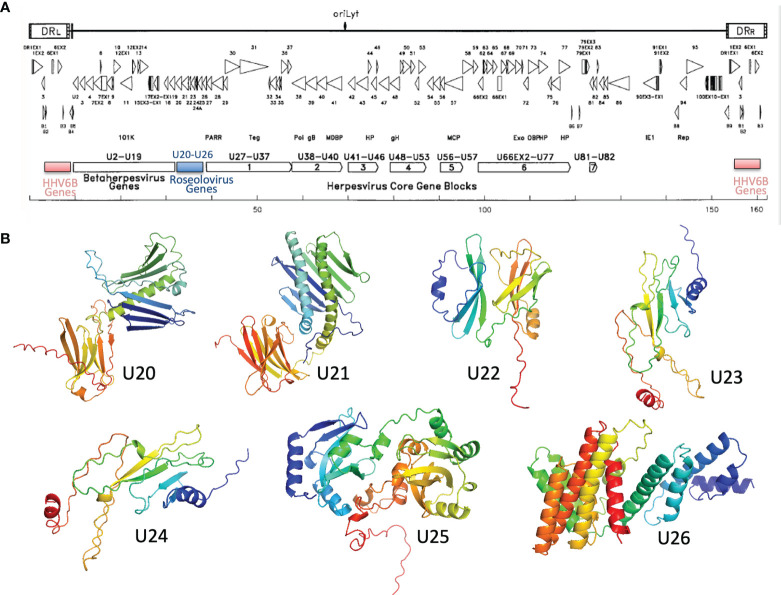
Roseolovirus-specific U20-U26 gene cluster. **(A)** Schematic diagram of HHV-6B gene organization, modified from (52). The U20-U26 cluster of genes specific to roseolovirus is indicated in blue. **(B)** AlphaFold predictions for members of the HHV-6B U20-U26 gene cluster.

U20-U26 comprise a gene cluster specific to the Roseolovirus genus, sandwiched between clusters of genes shared within the betaherpesvirus subfamily or by the entire herpesvirus family (**Figure 6A**) (52, 65). Generally, these genes are dispensable for viral growth (66) and involved in immune evasion: in addition to U20 and U21 described above, U24 has been implicated in endocytic recycling and protein degradation (67–69) and U26 has been shown to inhibit the RLR/MAVS signaling pathway (70). U20, U21, U23, and U24 are single-pass type I membrane glycoproteins, U25 is a soluble tegument protein, and U26 is a polytopic 8-pass integral membrane protein. To help evaluate the specificity of MHC-like fold prediction for membrane glycoproteins, and to identify possible additional MHC-like or immunoglobulin-like proteins in this gene cluster, we examined AlphaFold structures for the entire HHV-6B U20-U26 gene cluster, after removal of any predicted signal sequence and C-terminal membrane sequences. HHV-6B U21 was predicted to have an N-terminal MHC-platform domain and a C-terminal immunoglobulin-like domain, similarly to HHV-6B U20 but with the domains oriented somewhat differently. This will be discussed in detail in the next section. None of the predicted structures for U22-U26 contain immunoglobulin-like or MHC-like domains, although beta sheets and helices are apparent, indicating substantial specificity in prediction of MHC-like folds (**Figure 6B**).

## AlphaFold and RoseTTAfold Structural Models for U20 and U21

**Figure 7:**
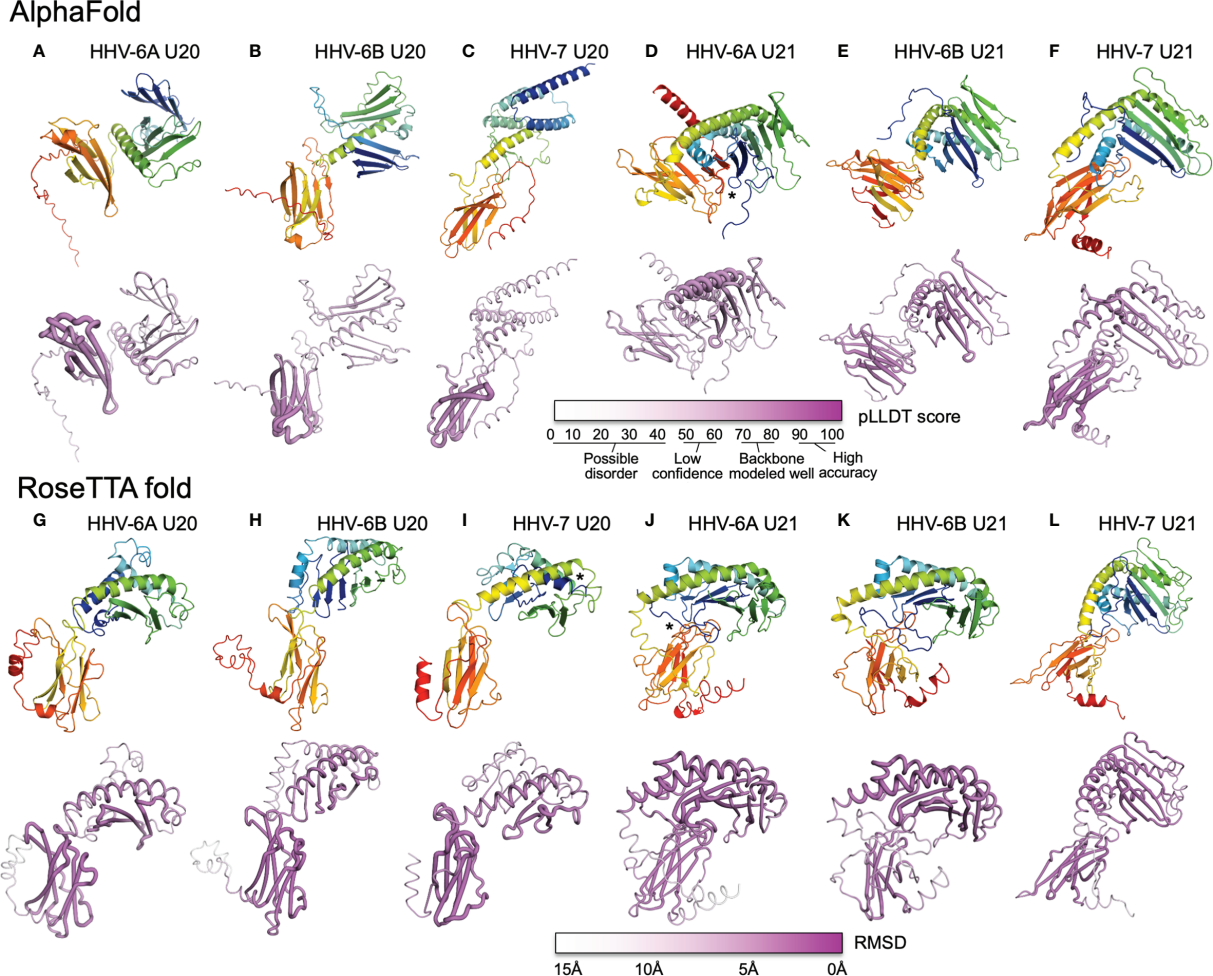
AlphaFold and RoseTTAfold structures for U20 and U21. AlphaFold structure predictions for U20 from HHV-6A **(A)**, HHV-6B **(B)**, and HHV-7 **(C)**, and for U21 from HHV-6A **(D)**, HHV-6B **(E)**, and HHV-7 **(F)**. RoseTTAfold structure predictions for U20 from HHV-6A **(G)**, HHV-6B **(H)**, and HHV-7 **(I)**, and for U21 from HHV-6A **(J)**, HHV-6B **(K)**, and HHV-7 **(L)**. The same sequences used in the alignments were processed through SignalP to identify signal sequences and TMHMM to identify transmembrane domains (73, 74). The sequences were then truncated to reflect the extracellular portion of the protein before use for structure prediction. For Phyre2, sequences were submitted for analysis *via* the Phyre2 server utilizing the “Intensive” modeling mode (57). For I-TASSER, we provided a protein sequence and specified no constraints or template exclusions (59). For AlphaFold we used the AlphaFold2 Advanced Script hosted by Google Colab (23). We used the default settings, specifically utilizing *de novo* generation of multisequence alignments with mmseqs2. We generated 5 models for each prediction with 1 ensemble, 3 recycles, a tolerance of 0, and 1 random seed. For RoseTTAfold we used the Robetta server and predicted on a single sequence using the “RoseTTAFold” method with no additional constraints (24). Both AlphaFold2 and RoseTTAFold generate several structural models from each modeling run. Figures were generated using the top-scoring model. For AlphaFold and RoseTTAfold, the top five scoring models from each run were examine for consistency with the model presented. Scale bars representing the confidence intervals are shown on a linear scale, RMSD for Phye2 and RoseTTA, TM-score for ITASSER, and pLDDT for AlphaFold. **(A-L)** Predicted structural models and previously determined crystal structures were visualized and figures were prepared using the Pymol molecular graphics program (75). Ribbon and tube diagrams colored as in **Figure 5**. Asterisks indicate features highlighted in text.

We used AlphaFold and RoseTTAfold to predict three-dimensional structures for U20 and U21 from each of the three human roseoloviruses HHV-6A, HHV-6B, and HHV-7. DeepMind and the European Bioinformatics Institute are developing an extensive, openly accessible database intended to eventually include AlphaFold predictions for most or all annotated genomes, but at the current time viral sequences are not included (71). We used AlphaFold2 Advanced CoLab, a Google-based computational environment, and Robetta, a distributed computing project hosted by the Baker laboratory at University of Washington, HHMI, and Rosetta@home, to provide access to RoseTTAfold (72). The HHV-6A and HHV-6B versions of U20 and U21 have high sequence homology (91% and 90% respectively (**Figure 6**), likely indicating very similar three-dimensional structures. We predicted structures for each separately because in some cases they have been shown to have different functional effects and binding specificities, as noted above, and as a test of the sensitivity of the folding algorithms to sequence variation. Predicted structures are shown as ribbon diagrams colored according to the linear sequence from N-terminus (blue) to C-terminus (red), and in earlier figures. Confidence scores along the sequences are shown in tube diagrams in the lower portion of each panel (magenta). For the pLDDT score provided by AlphaFold (**Figures 7A**–**F**), values of 90-100 indicate regions modeled with high accuracy including side-chain conformations, 70-80 indicated regions of less confidence for which the backbone is expected to be modeled well, and 50-60 indicate values of low confidence. Values below 50 indicate regions of possible disorder, for which there is no confidence in the structural prediction (76). RoseTTAfold provides a conventional RMSD score to characterize the Cα variation among predicted versions of the same structural regions; we converted this to a linear score running from 15Å (no confidence) to 0Å (high confidence) (**Figures 7G**–**L**). Both AlphaFold and RoseTTAfold provide a set of models that represent the structure prediction. In **Figure 7** we show the top scoring model for each prediction run, but in each case, we examined the top five models for structural consistency. In general, the top five scoring models were similar, with differences restricted to the low-confidence and likely unfolded regions at the proteins’ extreme N- and C-termini, and to differences between the relative orientation of the MHC-like and immunoglobulin-like domains. In a few cases, for the lower-scoring models, pairs of strands were separated from the main beta sheets, but as these were not consistently observed in multiple models we did not consider them further.

Each of the U20 and U21 proteins was predicted to adopt an MHC-Ib-like fold, with an immunoglobulin-like domain close to the C-terminus, and a characteristic MHC platform domain consisting of two α-helices atop an eight-stranded beta-sheet platform (**Figures 7A**–**L**). In all cases the immunoglobulin-like domains were predicted with higher confidence, and the MHC platform domains with lower confidence, generally with α-helical regions predicted with higher confidence than the beta sheet, and at least some of the β-strands with low or minimal confidence. There were several alterations in the MHC platform conformations relative to canonical structures, as occasionally observed for other MHC-Ib proteins. In the AlphaFold predictions of HHV-6A U20 (**Figure 7A**) and HHV-6B U20 (**Figure 7B**), the MHC platforms were the most distorted relative to a canonical MHC fold, with the α1 and α2 components of the MHC platform separated from each other for HHV-6A U20 and the α1 helical domain unfolded for HHV-6B U20. The RoseTTAfold predictions for these proteins (**Figures 7G, H**) had more standard MHC platform domains, although with some α1 strands missing for HHV-6B U20. All but one of the predicted structures for the U20 and U21 proteins had α-helices closely together and aligned to form closed “binding sites,” as generally found in MHC1b proteins that do not present peptides or other ligands. The one exception was the RoseTTAfold structure for HHV-7 U20 (**Figure 7I**), for which the helices were separated at the end of the site where peptides conventionally bind in MHC-Ia proteins, and in fact the extreme N-terminal 15 residues formed a helix that docked in this region (asterisk in **Figure 7I**). However, a comparable structure was not seen in the AlphaFold version. For three of the predictions, RoseTTAfold HHV-6A U20 (**Figure 7G**), AlphaFold HHV-7 U20 (**Figure 7C**) and AlphaFold HHV-7 U21 (**Figure 7F**), some of the β-strands were missing from the α1-domain platform that usually comprises a 4-stranded beta sheet, and for one of predictions, AlphaFold HHV-6B U21 (**Figure 7E**), the immunoglobulin domain had nine instead of the canonical 7 β-strands.

In general, the relative orientation of the MHC platform and immunoglobulin-like domain varies widely between different proteins that adopt the MHC-like fold, ranging from almost perpendicular for classical MHC-Ia proteins (such as H-2Kb in **Figure 3A**) to almost co-linear for some MHC-Ib proteins such as MICB (**Figure 3E**). We examined the angle between the MHC platform beta sheet and the immunoglobulin-like domain in each of our predicted U20 and U21 models and observed a range of interdomain orientations (**Figure 7**). The most extreme was for the AlphaFold U20 structures from both HHV-6A and HHV-6B (**Figures 7A, B**), for which the MHC platforms were flipped upside-down relative to the canonical MHC-Ia orientation. The RoseTTAfold structures for these proteins were somewhat more conventional, with the MHC platforms oriented roughly perpendicular to the immunoglobulin-like domain, but with the platforms swung out to the side with an extended linker between the domains and essentially no interdomain contacts (**Figures 7G, H**). These features might be indicative of substantial interdomain flexibility for U20 from HHV-6A and -6B. For U20 from HHV-7 (**Figures 7C, I**), the domains were roughly perpendicular, with a small area of contact between the top of immunoglobulin-like domain and loops between the strands in the MHC platform, apparent in both AlphaFold (**Figure 7C**) and RoseTTAfold (**Figure 7I**) models. The U21 models in general had more acute angles between MHC platform and immunoglobulin domain that for U20, in most cases with extensive contacts between the domains (**Figures 7D**–**F**, **J**–**L**). Each of the U21 models has a kinked C-terminal extension in the α2-domain α-helix, which orients the C-terminal end of the helix down towards the immunoglobulin-like domain. This same feature is also seen for the MCMV protein m152 (**Figure 3G**) as well as m153, m144, and m157 (31). For both AlphaFold and RoseTTAfold models of HHV-6A U21 (**Figures 7D, J**), the immunoglobulin-like domain is tucked underneath the MHC platform, with additional contacts from the extended N-terminal tails (asterisks in **Figures 7D, J**). However, the AlphaFold model (**Figure 7D**) has the immunoglobulin domain to the side of the MHC platform, still making extensive interdomain contacts, rather than underneath, as for the RoseTTAfold model (**Figure 7J**). For HHV-6B U21, both AlphaFold (**Figure 7E**) and RoseTTAfold (**Figure 7K**) models are quite similar to the corresponding models for HHV-6A U21 (**Figures 7D, J**). Finally, both models for HHV-7 U21 orient the immunoglobulin domain so that its edge makes extensive contacts with the underside of the MHC platform (**Figures 7F, G**).

Despite these variations from canonical structures, we considered the predictions of an MHC-Ib-like fold for each of the U20 and U21 proteins to be robust for two reasons. First, proteins with MHC-like folds generally contain two disulfide bonds, one between a cysteine residue in the α2-domain α-helix and another cysteine residue in a β-strand near the center of the MHC platform beta sheet as well as a canonical inter-sheet disulfide bond in the center of the immunoglobulin-like domain. The U20 extracellular domains have four (HHV-6A and -6B) or five (HHV-7) cysteines. In all the predicted U20 structures the cysteines are in position and modeled to form the expected disulfide bonds in the MHC platform and the immunoglobulin domain. The U21 extracellular domains contain more cysteine residues, six for HHV-6A, nine for HHV-6B, and 10 for HHV-7. In all the predicted U21 structures, the expected disulfide bonds in the MHC platform and immunoglobulin-like domains are formed, with an additional disulfide bond in the immunoglobulin domain in all U21 structures, and up to two additional disulfide bonds in the MHC platform for HHV-6B and HHV-7 U21. The expected disulfide bonding pattern is not known to the folding algorithms, and so formation of the typical MHC-fold disulfides represents an independent confirmation of the MHC-Ib-like fold. Second, U20 extracellular domains have a large number of potential N-linked glycosylation sites: nine for HHV-6A and HHV-6B at identical positions, and seven for HHV-7. For HHV-6B U20 we have verified that each of the sites is modified in recombinant soluble protein expressed in HEK-293 GnTI cells (GW and LJS, unpublished results). For each of the predicted U20 structures, the N-linked Asn residues are on the surface of the protein. U21 proteins have fewer N-linked glycosylation sites: one for HHV-6A, two for HHV-6B, and four for HHV-7 (77). For each of the predicted U21 structures, these glycosylation sites also are located on the protein surface. Surface accessibility of each of the asparagine residues involved in N-linked glycan formation provides some additional confidence in the overall model conformations.

We examined the predicted structures for compatibility with previously identified NK immunoevasin mechanisms involving MHC-Ia and MHC-Ib proteins for which structural information is available. U20 from HHV-6A recently has been reported to down-regulate the stress-induced MHC-platform-only MHC-Ib protein ULBP-1 (18). We investigated whether the predicted U20 structures and structural mechanisms from previous work would be consistent with this activity. The structure of ULBP-1 is not known, but ULBP-3 and ULBP-6 structures are highly similar to each other (29, 78) and to mouse RAE-1γ (31), a member of the murine RAE-1 family, which is orthologous to the human ULBP family. Previous structural work has shown that the m152 protein from MCMV binds to RAE-1γ, in a pincer-like mechanism that uses both the underside of its MHC platform and the edge of the its immunoglobulin domain to surrounding the top of the RAE-1γ MHC platform (31), as shown in **Figure 3E**. The structural models for U20 from HHV-6A, HHV-6B, and HHV-7 are all consistent with this mechanism, with no apparent steric interference for ULBP-1 underneath the platform for any of the models, and with immunoglobulin-like domains oriented appropriately for contacting ULBP-1 in the RoseTTAfold models. For MCMV m152, the interaction with RAE-1 results in down-regulation of RAE-1 surface expression, primarily *via* retention within the early secretory pathway (79). A similar interaction between U20 and ULBP-1 could explain the U20-mediated ULBP-1 down-regulation activity recently reported for HHV-6A. We note that this interaction would also be consistent with an MHC1b surface masking mechanism as recently proposed for m152 (32). Thus, the m152-RAE1-γ interaction potentially provides a model for U20 down-regulation of ULBP-1.

U21 from HHV-7 has been reported to down-regulate both nonclassical (MICA, MICB, HLA-E, and HLA-G) (18–20) and classical MHC-Ia proteins (15). We investigated whether the predicted U21 structures and previous structural characterization of other viral immunoevasins would be consistent with this activity. For MICA and MICB, the m152-RAE-1γ model just described for U20 would be consistent with the U21 structural predictions, although interactions with both the MHC platform and immunoglobulin-like domains would require some domain reorientation. Because classical MHC-Ia proteins have immunoglobulin-like domains and β2-microglobulin domains in addition the MHC platform, the m152-RAE-1γ model is not directly applicable to modeling classical MHC-Ia down-regulation, but could be relevant if the MHC-Ia protein adopted a supine conformation on the membrane (80). It is also possible that U21 uses distinct mechanisms to interact with MHC-Ia and MHC-Ib proteins. We considered whether previously reported structures of Ig-only viral immunoevasins US2 and CPXV203 (**Figures 3G, H**) could provide a model for the observed U21-mediated MHC-Ia down-regulation. The structural models for HHV-7 U21 would be consistent with the US2 mechanism without substantial steric interference, but for the CPXV203 mechanism the U21 MHC platform domains would interfere with the immunoglobulin-like domain docking on the MHC-Ia target. Finally, U20 from HHV-6B has been reported to interfere with TNFα signaling (48). The MHC-Ib protein 2L from poxvirus YDV provides a potential model for this activity (47, 81). The regions of the U20 structural models corresponding to those from YDL 2L that bind TNFα (**Figure 3J**) are surface exposed and potentially available for cytokine interaction, but we did not consider the predicted structures to be sufficiently accurate for docking or other structural modeling to evaluate this possibility in detail.

## Discussion

No experimental three-dimensional structure is available for any roseolovirus U20 or U21 protein. Cellular studies have revealed functional roles for these proteins in evasion of NK responses by interference with surface expression of classical MHC-Ia and non-classical MHC-Ib proteins. Structural information on these proteins would help to define mechanisms for the interference, suggest potential binding partners, and contribute to understanding the basis for observed differences in activities for the HHV-6A, HHV-6B and HHV-7 orthologs of U20 and U21. Previous sequence analysis had suggested the presence of immunoglobulin-like domains and in some cases MHC-like domains for some of these proteins. We used the next-generation structure prediction algorithms encoded in the machine-learning programs AlphaFold and RoseTTAfold to predict three-dimensional structures for the extracellular domains of the U20 and U21 glycoproteins from HHV-6A, HHV-6B, and HHV-7. All proteins were predicted to adopt MHC-like folds characteristic of non-classical MHC-Ib proteins. Structural models for U20 from all three viruses had MHC platform domains displaced from the immunoglobulin-like domains, with missing or altered structural elements relative to canonical structures, particularly in case of the α1 domains. Structural models for U21 from all three viruses had MHC platform domains closely opposed to the immunoglobulin-like domains. The U21 MHC platform domains had conventionally oriented α-helices atop an eight-stranded β sheet, in each case with a kinked and extended α-2 domain α-helix as previously observed in structures of m152, m153, and m157 from MCMV (31, 44, 82). We present the U20 and U21 models as guides for hypothesis generation about potential mechanisms of viral interference in MHC-Ia and MHC-Ib pathways, and to help understand observed differences between the HHV-6A, HHV-6B, and HHV-7 variants. We look forward to comparison of the models presented with here with experimentally-determined structures when they become available. It has been noted that while in some cases the accuracy of AlphaFold-derived models appears to surpass that of experimental methods (23), this may not be the case for new protein folds, where high-resolution experimentally-determined structures of close structural homologs are not available (83). In these cases, the expected accuracy is much lower, and has been estimated to correspond roughly to a very low resolution (~4Å) structure determined by X-ray crystallography (83).

For the U20 structural models, the interdomain orientation appeared to less well-defined than for U21, and in some models parts of the MHC platform were missing, disordered, or displaced. This could reflect bona fide aspects of U20 structure or conformational lability, but it is also possible that the U20 MHC platform and/or interdomain interaction might be stabilized by a binding partner. In conventional class Ia MHC proteins, peptide binding stabilizes the MHC platform and interdomain orientation, with synergistic stabilization by β2-microglobulin (84, 85). However, we do not expect that U20 or U21 require β2-microglobulin or peptide to complete folding, as recombinant proteins that do not contain peptide or β2-microglobulin retain full biding activity, at least for HHV-6B U20 binding to ULBP-1 (GW and LJS, unpublished results) and HHV-7 U21 binding to the MHC-Ia molecule HLA-A2 (50). It is also possible that oligomer formation could stabilize U20 and U21 folding, and in solution both recombinant HHV-6B U20 and HHV-7 U21 form dimers (GW and LJS, unpublished results) or tetramers (50), respectively. To evaluate the possibility that dimer formation might stabilize U20 folding, we used AlphaFold-multimer (86) to predict structures for the HHV-6B U20 dimer. However, the resultant structural models did not reveal more well-ordered interactions within the MHC platform or between domains. Finally, U20 is heavily glycosylated, with the nine N-linked glycans representing ~35-40% of the apparent molecular weight of the extracellular portion as assessed by SDS-PAGE (CS and AWH, unpublished results). For some glycoproteins, N-linked glycans are required for proper protein folding (87–89), but the influence of these bound glycans is not included in the AlphaFold and RoseTTAfold algorithms (90). However, fully deglycosylated recombinant HHV-6B U20 exhibits no tendency to aggregate, with a thermal denaturation at ~60-62°C (GW and LJS, unpublished observations), similar to recombinant cMHC-I proteins (91), and we do not expect that the U20 glycans are required for adoption or stabilization of the folded structure.

One consistent feature of the prediction efforts reported here is that the structural models produced by RoseTTAfold are more compact than those produced by AlphaFold. RoseTTAfold models had fewer unstructured regions, fewer broken helices and sheets, fewer displaced secondary structure elements, and more interdomain contacts than did the corresponding AlphaFold models. The lack of more complete correspondence between the algorithms is puzzling. RoseTTAfold was designed as a simplified version of AlphaFold suitable for use with limited computational power, and generally thought to be slightly less accurate (24, 92). Both AlphaFold and RoseTTAfold rely on aligned sets of evolutionarily related sequence variants for co-variation analysis, but U20 and U21 do not have easily-identified orthologs outside of the roseolovirus family and even there sequence coverage is thin. It is possible that AlphaFold is more reliant than RoseTTAfold on these alignments and unable to fold portions of the structures because of the limited sequence coverage, or that the full DeepMind prediction engine would fold these regions more completely than the slightly limited Google CoLab implementation that we used. However, is also possible that these regions are in fact more structurally labile, and that RoseTTAfold is overzealous in packing and overoptimistic in its confidence calculations. It will be interesting to compare the predicted structural models presented here with experimental models for U20 and U21 to evaluate these possibilities.

There are several limitations to our study. Structural modeling approaches based on machine learning multi-track algorithms are very new, and confidence estimates derived from predictions of newly determined crystal structures and from cross-validation of PDB entries might overestimate the prediction accuracy, especially for proteins with novel folds, folds not well-represented in the database, or containing unstructured regions. We did not consider chaperones or binding partners that might be necessary to complete folding, nor attempt to model immunoevasion mechanisms for which there is no current structural model. Finally, we focused on U20 and U21 because these proteins have been implicated in immunoevasion mechanisms that in other viruses involve MHC-Ia proteins, but there might be additional non-classical MHC or other proteins expressed by roseoloviruses required to understand the full picture of roseolovirus MHC-Ia immunoevasion.

In conclusion, we evaluated structures for the extracellular domains of the U20 and U21 immunoevasin proteins from human roseoloviruses HHV-6A, HHV-6B and HHV-7, produced by recently described state-of-the-art machine-learning prediction engines. The expected relatively low accuracy of the structural models limited detailed interpretation, and we considered only backbone conformations. Despite this restriction, each of the proteins was confidently predicted to adopt an MHC-like fold with a closed MHC platform domain above a canonical immunoglobulin-like domain. Predicted conformational differences between U20 and U21 included missing or unstructured elements in the MHC platform α1 domain for the U20 proteins, and more substantial interaction between MHC platform and immunoglobulin domains for the U21 proteins.

## Data Availability Statement

The datasets presented in this study can be found in online repositories. The names of the repository/repositories and accession number(s) can be found in the article/supplementary material.

## Author Contributions

GW and LS conceived the project and performed analyses. CS and AH contributed information on U20 and U21 stability, glycosylation, and binding partners. GW, RA, and LS prepared the manuscript. All authors contributed to the article and approved the submitted version.

## Funding

This work was supported by NIH grants U19-AI109858 (LJS), R01-AI153828 (LJS) and T32-AI007349 (GW).

## Conflict of Interest

The authors declare that the research was conducted in the absence of any commercial or financial relationships that could be construed as a potential conflict of interest.

## Publisher’s Note

All claims expressed in this article are solely those of the authors and do not necessarily represent those of their affiliated organizations, or those of the publisher, the editors and the reviewers. Any product that may be evaluated in this article, or claim that may be made by its manufacturer, is not guaranteed or endorsed by the publisher.

## References

[1] Kumar S Stecher G Li M Knyaz C Tamura K MEGA X. Molecular Evolutionary Genetics Analysis Across Computing Platforms. Mol Biol Evol (2018) 35:1547–9. doi: 10.1093/molbev/msy096 PMC5967553 29722887

[2] Hall CB Long CE Schnabel KC Caserta MT McIntyre KM Costanzo MA. Human Herpesvirus-6 Infection in Children. A Prospective Study of Complications and Reactivation. New Engl J Med (1994) 331:432–8. doi: 10.1056/NEJM199408183310703 8035839

[3] Huang LM Lee CY Liu MY Lee PI. Primary Infections of Human Herpesvirus-7 and Herpesvirus-6: A Comparative, Longitudinal Study Up to 6 Years of Age. Acta Paediatrica (1997) 86:604–8. doi: 10.1111/j.1651-2227.1997.tb08942.x 9202795

[4] Zerr DM Meier AS Selke SS Frenkel LM Huang M-L Wald A. A Population-Based Study of Primary Human Herpesvirus 6 Infection. New Engl J Med (2005) 352:768–76. doi: 10.1056/NEJMoa042207 15728809

[5] Yamanishi K Shiraki K Kondo T Okuno T Takahashi M Asano Y. Identification of Human Herpesvirus-6 as a Causal Agent for Exanthem Subitum. Lancet (1988) 331:1065–7. doi: 10.1016/S0140-6736(88)91893-4 2896909

[6] Asano Y Yoshikawa T Suga S Kobayashi I Nakashima T Yazaki T. Clinical Features of Infants With Primary Human Herpesvirus 6 Infection (Exanthem Subitum, Roseola Infantum). Pediatrics (1994) 93:104–8. doi: 10.1542/peds.93.1.104 8265302

[7] de Pagter PJ Schuurman R Meijer E van Baarle D Sanders EA Boelens JJ. Human Herpesvirus Type 6 Reactivation After Haematopoietic Stem Cell Transplantation. J Clin Virol: Off Publ Pan Am Soc Clin Virol (2008) 43:361–6. doi: 10.1016/j.jcv.2008.08.008 18829379

[8] Dulery R Salleron J Dewilde A Rossignol J Boyle EM Gay J. Early Human Herpesvirus Type 6 Reactivation After Allogeneic Stem Cell Transplantation: A Large-Scale Clinical Study. Biol Blood Marrow Transplant (2012) 18:1080–9. doi: 10.1016/j.bbmt.2011.12.579 22212513

[9] Arbuckle JH Medveczky MM Luka J Hadley SH Luegmayr A Ablashi D. The Latent Human Herpesvirus-6A Genome Specifically Integrates in Telomeres of Human Chromosomes *In Vivo* and In Vitro. Proc Natl Acad Sci U States America (2010) 107:5563–8. doi: 10.1073/pnas.0913586107 PMC2851814 20212114

[10] Arbuckle JH Pantry SN Medveczky MM Prichett J Loomis KS Ablashi D. Mapping the Telomere Integrated Genome of Human Herpesvirus 6A and 6B. Virology (2013) 442:3–11. doi: 10.1016/j.virol.2013.03.030 23648233 PMC3696530

[11] Hudson AW. Roseoloviruses and Their Modulation of Host Defenses. Curr Opin Virol (2014) 9:178–87. doi: 10.1016/j.coviro.2014.09.009 25462451

[12] Adams EJ Luoma AM. The Adaptable Major Histocompatibility Complex (MHC) Fold: Structure and Function of Nonclassical and MHC Class I-Like Molecules. Annu Rev Immunol (2013) 31:529–61. doi: 10.1146/annurev-immunol-032712-095912 23298204

[13] Revilleza MJ Wang R Mans J Hong M Natarajan K Margulies DH. How the Virus Outsmarts the Host: Function and Structure of Cytomegalovirus MHC-I-Like Molecules in the Evasion of Natural Killer Cell Surveillance. J Biomed Biotechnol (2011) 2011:724607. doi: 10.1155/2011/724607 21765638 PMC3134397

[14] Halenius A Gerke C Hengel H. Classical and non-Classical MHC I Molecule Manipulation by Human Cytomegalovirus: So Many Targets-But How Many Arrows in the Quiver? Cell Mol Immunol (2015) 12:139–53. doi: 10.1038/cmi.2014.105 PMC4654289 25418469

[15] Hudson AW Howley PM Ploegh HL. A Human Herpesvirus 7 Glycoprotein, U21, Diverts Major Histocompatibility Complex Class I Molecules to Lysosomes. J Virol (2001) 75:12347–58. doi: 10.1128/JVI.75.24.12347-12358.2001 PMC116131 11711625

[16] Hirata Y Kondo K Yamanishi K. Human Herpesvirus 6 Downregulates Major Histocompatibility Complex Class I in Dendritic Cells. J Med Virol (2001) 65:576–83. doi: 10.1002/jmv.2075 11596096

[17] Schmiedel D Tai J Levi-Schaffer F Dovrat S Mandelboim O. Human Herpesvirus 6b Downregulates Expression of Activating Ligands During Lytic Infection To Escape Elimination by Natural Killer Cells. J Virol (2016) 90:9608–17. doi: 10.1128/JVI.01164-16 PMC5068514 27535049

[18] Chaouat AE Seliger B Mandelboim O Schmiedel D. The HHV-6a Proteins U20 and U21 Target NKG2D Ligands to Escape Immune Recognition. Front Immunol (2021) 12:714799. doi: 10.3389/fimmu.2021.714799 34721381 PMC8554080

[19] May NA Glosson NL Hudson AW. Human Herpesvirus 7 U21 Downregulates Classical and Nonclassical Class I Major Histocompatibility Complex Molecules From the Cell Surface. J Virol (2010) 84:3738–51. doi: 10.1128/JVI.01782-09 PMC2849505 20106916

[20] Schneider CL Hudson AW. The Human Herpesvirus-7 (HHV-7) U21 Immunoevasin Subverts NK-Mediated Cytoxicity Through Modulation of MICA and MICB. PloS Pathog (2011) 7:e1002362. doi: 10.1371/journal.ppat.1002362 22102813 PMC3213103

[21] Hudson AW Blom D Howley PM Ploegh HL. The ER-Lumenal Domain of the HHV-7 Immunoevasin U21 Directs Class I MHC Molecules to Lysosomes. Traffic (Copenhagen Denmark) (2003) 4:824–37. doi: 10.1046/j.1398-9219.2003.0137.x 14617346

[22] Glosson NL Hudson AW. Human Herpesvirus-6A and -6B Encode Viral Immunoevasins That Downregulate Class I MHC Molecules. Virology (2007) 365:125–35. doi: 10.1016/j.virol.2007.03.048 17467766

[23] Jumper J Evans R Pritzel A Green T Figurnov M Ronneberger O. Highly Accurate Protein Structure Prediction With AlphaFold. Nature (2021) 596:583–9. doi: 10.1038/s41586-021-03819-2 PMC8371605 34265844

[24] Baek M DiMaio F Anishchenko I Dauparas J Ovchinnikov S Lee GR. Accurate Prediction of Protein Structures and Interactions Using a Three-Track Neural Network. Science (2021) 373:871–6. doi: 10.1126/science.abj8754 PMC7612213 34282049

[25] Dam J Guan R Natarajan K Dimasi N Chlewicki LK Kranz DM. Variable MHC Class I Engagement by Ly49 Natural Killer Cell Receptors Demonstrated by the Crystal Structure of Ly49C Bound to H-2K(B). Nat Immunol (2003) 4:1213–22. doi: 10.1038/ni1006 14595439

[26] Yang Z Bjorkman PJ. Structure of UL18, a Peptide-Binding Viral MHC Mimic, Bound to a Host Inhibitory Receptor. Proc Natl Acad Sci U States America (2008) 105:10095–100. doi: 10.1073/pnas.0804551105 PMC2465803 18632577

[27] Prod’homme V Griffin C Aicheler RJ Wang EC McSharry BP Rickards CR. The Human Cytomegalovirus MHC Class I Homolog UL18 Inhibits LIR-1+ But Activates LIR-1- NK Cells. J Immunol (Baltimore Md.: 1950) (2007) 178:4473–81. doi: 10.4049/jimmunol.178.7.4473 PMC2843079 17372005

[28] Wagner CS Ljunggren HG Achour A. Immune Modulation by the Human Cytomegalovirus-Encoded Molecule UL18, a Mystery Yet to be Solved. J Immunol (Baltimore Md.: 1950) (2008) 180:19–24. doi: 10.4049/jimmunol.180.1.19 18096997

[29] Radaev S Rostro B Brooks AG Colonna M Sun PD. Conformational Plasticity Revealed by the Cocrystal Structure of NKG2D and its Class I MHC-Like Ligand ULBP3. Immunity (2001) 15:1039–49. doi: 10.1016/S1074-7613(01)00241-2 11754823

[30] Lazear E Peterson LW Nelson CA Fremont DH. Crystal Structure of the Cowpox Virus-Encoded NKG2D Ligand OMCP. J Virol (2013) 87:840–50. doi: 10.1128/JVI.01948-12 PMC3554055 23115291

[31] Wang R Natarajan K Revilleza MJ Boyd LF Zhi L Zhao H. Structural Basis of Mouse Cytomegalovirus M152/Gp40 Interaction With RAE1γ Reveals a Paradigm for MHC/MHC Interaction in Immune Evasion. Proc Natl Acad Sci USA (2012) 109:E3578–87. doi: 10.1073/pnas.1214088109 PMC3529088 23169621

[32] Lis N Hein Z Ghanwat SS Ramnarayan VR Chambers BJ Springer S. The Murine Cytomegalovirus Immunoevasin Gp40/M152 Inhibits NKG2D Receptor RAE-1γ by Intracellular Retention and Cell Surface Masking. J Cell Sci (2021) 134. doi: 10.1242/jcs.257428 34085696

[33] Stempel M Chan B Juranić Lisnić V Krmpotić A Hartung J Paludan SR. The Herpesviral Antagonist M152 Reveals Differential Activation of STING-Dependent IRF and NF-κb Signaling and STING’s Dual Role During MCMV Infection. EMBO J (2019) 38. doi: 10.15252/embj.2018100983 PMC6396373 30696688

[34] Dunn C Chalupny NJ Sutherland CL Dosch S Sivakumar PV Johnson DC. Human Cytomegalovirus Glycoprotein UL16 Causes Intracellular Sequestration of NKG2D Ligands, Protecting Against Natural Killer Cell Cytotoxicity. J Exp Med (2003) 197:1427–39. doi: 10.1084/jem.20022059 PMC2193902 12782710

[35] Welte SA Sinzger C Lutz SZ Singh-Jasuja H Sampaio KL Eknigk U. Selective Intracellular Retention of Virally Induced NKG2D Ligands by the Human Cytomegalovirus UL16 Glycoprotein. Eur J Immunol (2003) 33:194–203. doi: 10.1002/immu.200390022 12594848

[36] Müller S Zocher G Steinle A Stehle T. Structure of the HCMV UL16-MICB Complex Elucidates Select Binding of a Viral Immunoevasin to Diverse NKG2D Ligands. PloS Pathog (2010) 6:e1000723. doi: 10.15252/embj.2018100983 20090832 PMC2797645

[37] Holmes MA Li P Petersdorf EW Strong RK. Structural Studies of Allelic Diversity of the MHC Class I Homolog MIC-B, a Stress-Inducible Ligand for the Activating Immunoreceptor NKG2D. J Immunol (Baltimore Md.: 1950) (2002) 169:1395–400. doi: 10.4049/jimmunol.169.3.1395 12133964

[38] Gewurz BE Gaudet R Tortorella D Wang EW Ploegh HL Wiley DC. Antigen Presentation Subverted: Structure of the Human Cytomegalovirus Protein US2 Bound to the Class I Molecule HLA-A2. Proc Natl Acad Sci USA (2001) 98:6794–9. doi: 10.1073/pnas.121172898 PMC34432 11391001

[39] McCoy WHT Wang X Yokoyama WM Hansen TH Fremont DH. Structural Mechanism of ER Retrieval of MHC Class I by Cowpox. PloS Biol (2012) 10:e1001432. doi: 10.1371/journal.pbio.1001432 23209377 PMC3507924

[40] Li P Willie ST Bauer S Morris DL Spies T Strong RK. Crystal Structure of the MHC Class I Homolog MIC-A, a Gammadelta T Cell Ligand. Immunity (1999) 10:577–84. doi: 10.1016/S1074-7613(00)80057-6 10367903

[41] Rahim MM Makrigiannis AP. Ly49 Receptors: Evolution, Genetic Diversity, and Impact on Immunity. Immunol Rev (2015) 267:137–47. doi: 10.1111/imr.12318 26284475

[42] Deng L Cho S Malchiodi EL Kerzic MC Dam J Mariuzza RA. Molecular Architecture of the Major Histocompatibility Complex Class I-Binding Site of Ly49 Natural Killer Cell Receptors. J Biol Chem (2008) 283:16840–9. doi: 10.1074/jbc.M801526200 PMC2423261 18426793

[43] Tormo J Natarajan K Margulies DH Mariuzza RA. Crystal Structure of a Lectin-Like Natural Killer Cell Receptor Bound to its MHC Class I Ligand. Nature (1999) 402:623–31. doi: 10.1038/45170 10604468

[44] Adams EJ Juo ZS Venook RT Boulanger MJ Arase H Lanier LL. Structural Elucidation of the M157 Mouse Cytomegalovirus Ligand for Ly49 Natural Killer Cell Receptors. Proc Natl Acad Sci USA (2007) 104:10128–33. doi: 10.1073/pnas.0703735104 PMC1891256 17537914

[45] Corbett AJ Coudert JD Forbes CA Scalzo AA. Functional Consequences of Natural Sequence Variation of Murine Cytomegalovirus M157 for Ly49 Receptor Specificity and NK Cell Activation. J Immunol (Baltimore Md.: 1950) (2011) 186:1713–22. doi: 10.4049/jimmunol.1003308 21187440

[46] Berry R Ng N Saunders PM Vivian JP Lin J Deuss FA. Targeting of a Natural Killer Cell Receptor Family by a Viral Immunoevasin. Nat Immunol (2013) 14:699–705. doi: 10.1038/ni.2605 23666294

[47] Yang Z West AP Jr Bjorkman PJ. Crystal Structure of TNFalpha Complexed With a Poxvirus MHC-Related TNF Binding Protein. Nat Struct Mol Biol (2009) 16:1189–91. doi: 10.1038/nsmb.1683 PMC2819277 19838188

[48] Kofod-Olsen E Ross-Hansen K Schleimann MH Jensen DK Møller JML Bundgaard B. U20 Is Responsible for Human Herpesvirus 6b Inhibition of Tumor Necrosis Factor Receptor-Dependent Signaling and Apoptosis. J Virol (2012) 86:11483–92. doi: 10.1128/JVI.00847-12 PMC3486335 22896603

[49] Madeira F Park YM Lee J Buso N Gur T Madhusoodanan N. The EMBL-EBI Search and Sequence Analysis Tools APIs in 2019. Nucleic Acids Res (2019) 47:W636–41. doi: 10.1093/nar/gkz268 PMC6602479 30976793

[50] May NA Wang Q Balbo A Konrad SL Buchli R Hildebrand WH. Human Herpesvirus 7 U21 Tetramerizes to Associate With Class I Major Histocompatibility Complex Molecules. J Virol (2014) 88:3298–308. doi: 10.1128/JVI.02639-13 PMC3957921 24390327

[51] Reginster M Nermut MV. Preparation and Characterization of Influenza Virus Cores. J Gen Virol (1976) 31:211–20. doi: 10.1099/0022-1317-31-2-211 932691

[52] Dominguez G Dambaugh TR Stamey FR Dewhurst S Inoue N Pellett PE. Human Herpesvirus 6b Genome Sequence: Coding Content and Comparison With Human Herpesvirus 6a. J Virol (1999) 73:8040–52. doi: 10.1128/JVI.73.10.8040-8052.1999 PMC112820 10482553

[53] Cuff JA Clamp ME Siddiqui AS Finlay M Barton GJ. JPred: A Consensus Secondary Structure Prediction Server. Bioinformatics (1998) 14:892–3. doi: 10.1093/bioinformatics/14.10.892 9927721

[54] Smith HR Heusel JW Mehta IK Kim S Dorner BG Naidenko OV. Recognition of a Virus-Encoded Ligand by a Natural Killer Cell Activation Receptor. Proc Natl Acad Sci USA (2002) 99:8826–31. doi: 10.1073/pnas.092258599 PMC124383 12060703

[55] Kelley LA MacCallum RM Sternberg MJ. Enhanced Genome Annotation Using Structural Profiles in the Program 3D-PSSM. J Mol Biol (2000) 299:499–520. doi: 10.1006/jmbi.2000.3741 10860755

[56] Kelley LA Sternberg MJ. Protein Structure Prediction on the Web: A Case Study Using the Phyre Server. Nat Protoc (2009) 4:363–71. doi: 10.1038/nprot.2009.2 19247286

[57] Kelley LA Mezulis S Yates CM Wass MN Sternberg MJ. The Phyre2 Web Portal for Protein Modeling, Prediction and Analysis. Nat Protoc (2015) 10:845–58. doi: 10.1038/nprot.2015.053 PMC5298202 25950237

[58] Zhang Y. I-TASSER Server for Protein 3D Structure Prediction. BMC Bioinf (2008) 9:40. doi: 10.1186/1471-2105-9-40 PMC2245901 18215316

[59] Yang J Yan R Roy A Xu D Poisson J Zhang Y. The I-TASSER Suite: Protein Structure and Function Prediction. Nat Methods (2015) 12:7–8. doi: 10.1038/nmeth.3213 25549265 PMC4428668

[60] Sample I. Google’s DeepMind Predicts 3D Shapes of Proteins. The Guardian (2018).

[61] Silver D Hubert T Schrittwieser J Antonoglou I Lai M Guez A. A General Reinforcement Learning Algorithm That Masters Chess, Shogi, and Go Through Self-Play. Science (2018) 362:1140–4. doi: 10.1126/science.aar6404 30523106

[62] Moult J. Predicting Protein Three-Dimensional Structure. Curr Opin Biotechnol (1999) 10:583–8. doi: 10.1016/S0958-1669(99)00037-3 10600698

[63] Senior AW Evans R Jumper J Kirkpatrick J Sifre L Green T. Protein Structure Prediction Using Multiple Deep Neural Networks in the 13th Critical Assessment of Protein Structure Prediction (Casp13). Proteins: Struct Function Bioinf (2019) 87:1141–8. doi: 10.1002/prot.25834 PMC7079254 31602685

[64] Mariani V Biasini M Barbato A Schwede T. lDDT: A Local Superposition-Free Score for Comparing Protein Structures and Models Using Distance Difference Tests. Bioinformatics (2013) 29:2722–8. doi: 10.1093/bioinformatics/btt473 PMC3799472 23986568

[65] Nicholas J. Determination and Analysis of the Complete Nucleotide Sequence of Human Herpesvirus. J Virol (1996) 70:5975–89. doi: 10.1128/jvi.70.9.5975-5989.1996 PMC190618 8709220

[66] Jasirwan C Tang H Kawabata A Mori Y. The Human Herpesvirus 6 U21–U24 Gene Cluster is Dispensable for Virus Growth. Microbiol Immunol (2015) 59:48–53. doi: 10.1111/1348-0421.12208 25346365

[67] Sullivan BM Coscoy L. Downregulation of the T-Cell Receptor Complex and Impairment of T-Cell Activation by Human Herpesvirus 6 U24 Protein. J Virol (2008) 82:602–8. doi: 10.1128/JVI.01571-07 PMC2224597 17977973

[68] Sullivan BM Coscoy L. The U24 Protein From Human Herpesvirus 6 and 7 Affects Endocytic Recycling. J Virol (2010) 84:1265–75. doi: 10.1128/JVI.01775-09 PMC2812311 19923186

[69] Sang Y Zhang R Creagh AL Haynes CA Straus SK. Interactions of U24 From Roseolovirus With WW Domains: Canonical vs Noncanonical. Biochem Cell Biol (2017) 95:350–8. doi: 10.1139/bcb-2016-0250 28314105

[70] Jiang X Tang T Guo J Wang Y Li P Chen X. Human Herpesvirus 6b U26 Inhibits the Activation of the RLR/MAVS Signaling Pathway. mBio (2021) 12. doi: 10.1128/mBio.03505-20 PMC8545120 33593967

[71] Varadi M Anyango S Deshpande M Nair S Natassia C Yordanova G. AlphaFold Protein Structure Database: Massively Expanding the Structural Coverage of Protein-Sequence Space With High-Accuracy Models. Nucleic Acids Res (2021) 50:D439–44. doi: 10.1093/nar/gkab1061 PMC8728224 34791371

[72] Baek M DiMaio F Anishchenko I Dauparas J Ovchinnikov S Lee GR. Accurate Prediction of Protein Structures and Interactions Using a 3-Track Network. bioRxiv (2021). 2021.06.14.448402. doi: 10.1101/2021.06.14.448402 PMC7612213 34282049

[73] Sonnhammer EL von Heijne G Krogh A. A Hidden Markov Model for Predicting Transmembrane Helices in Protein Sequences. Proc Int Conf Intelligent Syst Mol Biol (1998) 6:175–82. 9783223

[74] Almagro Armenteros JJ Tsirigos KD Sønderby CK Petersen TN Winther O Brunak S. SignalP 5.0 Improves Signal Peptide Predictions Using Deep Neural Networks. Nat Biotechnol (2019) 37:420–3. doi: 10.1038/s41587-019-0036-z 30778233

[75] Schrodinger LLC. The PyMOL Molecular Graphics System, Version 1.8. (2015).

[76] EMBL-EBI. Frequently Asked Questions - AlphaFold Protein Structure Database. Wellcome Genome Campus (2021).

[77] Glosson NL Gonyo P May NA Schneider CL Ristow LC Wang Q. Insight Into the Mechanism of Human Herpesvirus 7 U21-Mediated Diversion of Class I MHC Molecules to Lysosomes. J Biol Chem (2010) 285:37016–29. doi: 10.1074/jbc.M110.125849 PMC2978630 20833720

[78] Zuo J Willcox CR Mohammed F Davey M Hunter S Khan K. A Disease-Linked ULBP6 Polymorphism Inhibits NKG2D-Mediated Target Cell Killing by Enhancing the Stability of NKG2D Ligand Binding. Sci Signaling (2017) 10:eaai8904. doi: 10.1126/scisignal.aai8904 28559451

[79] Arapovic J Lenac T Antulov R Polic B Ruzsics Z Carayannopoulos LN. Differential Susceptibility of RAE-1 Isoforms to Mouse Cytomegalovirus. J Virol (2009) 83:8198–207. doi: 10.1128/JVI.02549-08 PMC2715744 19494006

[80] Mitra AK Célia H Ren G Luz JG Wilson IA Teyton L. Supine Orientation of a Murine MHC Class I Molecule on the Membrane Bilayer. Curr Biol (2004) 14:718–24. doi: 10.1016/j.cub.2004.04.004 15084288

[81] Rahman MM Jeng D Singh R Coughlin J Essani K McFadden G. Interaction of Human TNF and Beta2-Microglobulin With Tanapox Virus-Encoded TNF Inhibitor, TPV-2l. Virology (2009) 386:462–8. doi: 10.1016/j.virol.2009.01.026 19232662

[82] Mans J Natarajan K Balbo A Schuck P Eikel D Hess S. Cellular Expression and Crystal Structure of the Murine Cytomegalovirus Major Histocompatibility Complex Class I-Like Glycoprotein, M153. J Biol Chem (2007) 282:35247–58. doi: 10.1074/jbc.M706782200 PMC2424207 17897947

[83] Moore PB Hendrickson WA Henderson R Brunger AT. The Protein-Folding Problem: Not Yet Solved. Science (2022) 375:507. doi: 10.1126/science.abn9422 35113705

[84] van Hateren A Bailey A Werner JM Elliott T. Plasticity of Empty Major Histocompatibility Complex Class I Molecules Determines Peptide-Selector Function. Mol Immunol (2015) 68:98–101. doi: 10.1016/j.molimm.2015.03.010 25818313 PMC4726658

[85] Jantz-Naeem N Springer S. Venus Flytrap or Pas De Trois? The Dynamics of MHC Class I Molecules. Curr Opin Immunol (2021) 70:82–9. doi: 10.1016/j.coi.2021.04.004 33993034

[86] Evans R O’Neill M Pritzel A Antropova N Senior A Green T. Protein Complex Prediction With AlphaFold-Multimer. bioRxiv (2021). 2021.10.04.463034. doi: 10.1101/2021.10.04.463034

[87] Hanson SR Culyba EK Hsu TL Wong CH Kelly JW Powers ET. The Core Trisaccharide of an N-Linked Glycoprotein Intrinsically Accelerates Folding and Enhances Stability. Proc Natl Acad Sci USA (2009) 106:3131–6. doi: 10.1073/pnas.0810318105 PMC2651298 19204290

[88] Tokhtaeva E Munson K Sachs G Vagin O. N-Glycan-Dependent Quality Control of the Na,K-ATPase Beta(2) Subunit. Biochemistry (2010) 49:3116–28. doi: 10.1021/bi100115a PMC3186216 20199105

[89] Mochizuki K Kagawa T Numari A Harris MJ Itoh J Watanabe N. Two N-Linked Glycans are Required to Maintain the Transport Activity of the Bile Salt Export Pump (ABCB11) in MDCK II Cells. Am J Physiol Gastrointest Liver Physiol (2007) 292:G818–28. doi: 10.1152/ajpgi.00415.2006 17082223

[90] Bagdonas H Fogarty CA Fadda E Agirre J. The Case for Post-Predictional Modifications in the AlphaFold Protein Structure Database. Nat Struct Mol Biol (2021) 28:869–70. doi: 10.1038/s41594-021-00680-9 34716446

[91] Hellman LM Yin L Wang Y Blevins SJ Riley TP Belden OS. Differential Scanning Fluorimetry Based Assessments of the Thermal and Kinetic Stability of Peptide-MHC Complexes. J Immunol Methods (2016) 432:95–101. doi: 10.1016/j.jim.2016.02.016 26906089 PMC4837003

[92] Akdel M Pires DEV Porta Pardo E Jänes J Zalevsky AO Mészáros B. A Structural Biology Community Assessment of AlphaFold 2 Applications. bioRxiv (2021). 2021.09.26.461876. doi: 10.1101/2021.09.26.461876

