# Corrigendum: Structural Models for Roseolovirus U20 And U21: Non-Classical MHC-I Like Proteins From HHV-6A, HHV-6B, and HHV-7

**DOI:** 10.3389/fimmu.2022.936968

**Published:** 2022-05-23

**Authors:** Grant C. Weaver, Richa Arya, Christine L. Schneider, Amy W. Hudson, Lawrence J. Stern

**Affiliations:** ^1^Immunology and Microbiology Graduate Program, Morningside Graduate School of Biomedical Sciences, UMass Chan Medical School, Worcester, MA, United States; ^2^Department of Pathology, UMass Chan Medical School, Worcester, MA, United States; ^3^Department of Life Sciences, Carroll University, Waukesha, WI, United States; ^4^Department of Microbiology and Molecular Genetics, Medical College of Wisconsin, Milwaukee, WI, United States; ^5^Department of Biochemistry and Molecular Biotechnology, UMass Chan Medical School, Worcester, MA, United States

**Keywords:** human herpesvirus, major histocompatibility protein, immunoevasion, machine learning, structure prediction, MHC1b, natural killer cell ligand, immune recognition

In the published article, there was an error regarding the affiliation for **Christine L. Schneider**. The correct affiliation should read “**Department of Life Sciences, Carroll University, Waukesha, WI, United States**”.

The authors apologize for this error and state that this does not change the scientific conclusions of the article in any way. The original article has been updated.

## Publisher’s Note

All claims expressed in this article are solely those of the authors and do not necessarily represent those of their affiliated organizations, or those of the publisher, the editors and the reviewers. Any product that may be evaluated in this article, or claim that may be made by its manufacturer, is not guaranteed or endorsed by the publisher.

